# Implementing the Empowering Better End of life Dementia Care (EMBED-Care) Framework to improve palliative dementia care in care homes: A feasibility evaluation

**DOI:** 10.1177/20552076261476137

**Published:** 2026-09-08

**Authors:** Juliet Gillam, Nathan Davies, Clare Ellis-Smith, Ayesha Dar, Elizabeth Sampson, Catherine Evans

**Affiliations:** 1 The Geller Institute of Aging and Memory, 7364 University of West London, United Kingdom; 2 Florence Nightingale Faculty of Nursing, Midwifery & Palliative Care, Cicely Saunders Institute, 4617 King’s College London, United Kingdom; 3 Centre for Psychiatry and Mental Health, Wolfson Institute of Population Health, 4616 Queen Mary University of London, United Kingdom; 4 Research Department of Primary Care and Population Health, University College London, London, United Kingdom; 5 Division of Psychiatry, University College London, United Kingdom 4919

**Keywords:** Implementation, dementia, care homes, eHealth

## Abstract

**Objective:**

Care homes are the main provider of palliative care for people living with dementia. In the UK around 70% of care homes are residential, with no onsite medical or nursing staff to manage residents’ complex needs. eHealth interventions can support holistic care delivery in this context; however, adoption in care homes has been limited. Innovative approaches grounded in implementation science are required to encourage uptake. This study aimed to assess the feasibility of a theoretically informed implementation plan for the EMBED-Care Framework, an eHealth intervention delivered via a mobile app to support holistic assessment and decision-making for people living with dementia in care homes.

**Methods:**

An embedded mixed-methods feasibility study was conducted in two residential homes. Qualitative data included non-participant observations, focus groups and semi-structured interviews with staff, analysed using codebook thematic analysis informed by Normalisation Process Theory. Quantitative data comprised app-usage metrics, analysed descriptively. Patient and public involvement informed the study design and conduct.

**Results:**

Twenty staff across two care homes used the intervention with 26 residents. Although some uptake was observed, usage data indicated that the Framework was largely not used as intended. Whilst care staff acknowledged its potential benefit, they faced substantial practical challenges to its use related to workload and a sense it was duplicating existing care processes. These issues led to negative appraisals of its impact and compromised staff engagement.

**Conclusion:**

Feasibility evaluation enabled rapid identification of barriers and refinement of implementation strategies. Iterative revisions to strategies related to providing further staff support, including a more tailored approach to training, and additional reminders to use the Framework. The Framework itself also requires adaptation to improve ‘fit’ in the care home context. Further collaboration on the revised strategies with people living with dementia and their family carers is required.

## Introduction

Palliative care requires a holistic, interdisciplinary approach to support people with life-limiting illness, addressing physical, psychological, social, and spiritual needs to enhance comfort and quality of life.^1^ In the UK, most palliative care for people with dementia is delivered in residential care homes.^2^ Residential homes provide personal care and support but do not employ onsite registered nurses, while nursing homes have registered nursing staff available to provide continuous nursing care alongside personal care. Strengthening staff capacity to manage complex needs within residential care homes is critical to reduce avoidable hospital admissions^3^ and support preferred place of death.^4^

The rapid expansion of eHealth - “health services and information delivered or enhanced through the internet and related technologies”^5^ - during the Covid-19 pandemic^6^ demonstrated its potential to improve assessment, symptom recognition, and decision-making in care homes, providing accessible tools for staff without formal healthcare training.^7^

Despite demonstrable advantages, adoption of eHealth has classically been faced with resistance^8^; changing routine practice is a time-consuming, complex process. The Covid-19 pandemic went some way to circumvent this, with universal recognition of the need for information sharing and conducting vital care processes virtually.^9^ However, as rapid practical action was required, little attention was paid to documenting the processes surrounding the implementation of eHealth.^10^

Challenges to implementing interventions in long-term care settings are well documented in the literature. Peryer and colleagues describe care homes as “complex adaptive systems of interconnected sub-systems where people, tasks, technologies, the physical environment and organisational culture interact”^11^; attempts to change routine practice in what are inherently complex settings will inevitably encounter a series of obstacles. Barriers commonly relate to staffing pressures including high turnover and perceptions of an unfeasible workload, and interventions which are poorly tailored to the needs of the specific care homes.^12^ Delegation of champions, thorough user-training and regular and on-going communication and input from the research team facilitate implementation, with recommendations often including a focus on addressing sustainability ahead of implementation, rather than retrospectively.^13^

In the research sphere there has been growing recognition of the importance of employing methods from implementation science, to close the frequently cited ‘17-year gap’ before research evidence reaches clinical practice.^14^ Evaluating implementation strategies - techniques used to enhance the adoption, implementation, and sustainability of an intervention^15^ - and identifying what influences whether they are successful in bringing about change in routine practice, is necessary to maximise the likelihood of their impact.^16^

### Aim

This study aimed to evaluate the feasibility of implementing an eHealth intervention - the EMBED-Care Framework - to improve holistic assessment and decision-making for people with dementia in care homes.

## Methods

Ethical approval was received by the HRA and Research Ethics Committee (REC) at Queen Square (REC Ref: 23/LO/0055).

### Design

An embedded mixed methods 16-week study, with a concurrent quantitative strand in a primarily qualitative design.

### Setting and participants

Two residential care homes in Greater London were recruited. The care homes were identified either through contacts at the NIHR Clinical Research Networks (CRN) for South London, Kent, Surrey and Sussex, North Thames, North West London, and Mid and South Essex, via the Enabling Research in Care Homes (ENRICH) network^17^ or via online searches of eligible care homes. Care homes were eligible if they were registered as a residential care home, with no on-site nursing provision, and had a minimum of 15 residents with either a formal diagnosis of dementia, or symptoms suggestive of cognitive decline. Only residential care homes were included in this study, as the IPOS-Dem was developed with and for social care staff, and the aim of the Framework is to facilitate health care delivery in care homes, for carers who may have no formal health care training.

A planned sample of 20 staff participants was estimated to establish feasibility and generate sufficient information power.^18^ Prior research has suggested that 10 users testing new technology is sufficient to identify 80% of issues, and 20 users to identify 95%.^19^ Staff were eligible if they provided direct care to residents, were able to read and write in English and had capacity to provide written informed consent, which was obtained from all the participants prior to study initiation.

The target group for the intervention was all individuals with dementia residing in the care homes. As it is estimated that 70% of individuals in care homes have dementia but many do not receive a diagnosis,^20^ a formal dementia diagnosis was not a requirement for eligibility. In absence of a diagnosis, a resident with symptoms suggestive of dementia, or a cognitive impairment resulting from dementia of any type or any severity, was considered eligible.

### The eHealth intervention

The EMBED-Care Framework, delivered via an app, was designed with the overarching intention of optimising palliative, person-centred end-of-life dementia care, developed as part of the EMBED-Care programme.^21^

The Framework comprises 1) The Integrated-Palliative care Outcome Scale (IPOS-Dem)^22^; a comprehensive 28-item person-centred outcome measure (PCOM) to facilitate identification of unmet needs to inform care priorities, linked with 2) decision support tools based on ‘rules of thumb’^23,24^ to which the user is directed to if a need is identified as unstable or unmet in order to promote some sort of action to improve care. Fortnightly assessment and review with each resident is advised, or more regularly for more complex or changing care needs or in the final days of life. Use of the intervention is supported by a 3-hour face-to-face training session, inbuilt training videos and resources on the app and ‘champions’; staff members delegated by the care home manager whose role it is to drive implementation of the EMBED-Care Framework by supporting other staff and initiating use of the Framework, to bring about a change in routine practice.

### Underpinning theory

This study forms the third and final component of a wider sequential study to develop and evaluate the implementation plan for the Framework.

Determinants of implementation of eHealth interventions in care homes, identified in a systematic review,^25^ were pursued in relation to the EMBED-Care Framework in a subsequent co-design study.^26^ Additional determinants were identified with participants, underpinned by Normalistion Process Theory (NPT).^27^ NPT identifies four key constructs which shape the social processes of implementation: (i) coherence (the work people do to make sense of a new intervention), (ii) cognitive participation (the work people do to engage with a new intervention), (iii) collective action (the work people do to enact a new intervention) and (iv) reflexive monitoring (the work people do to appraise a new intervention.^27^

**Figure 1.**
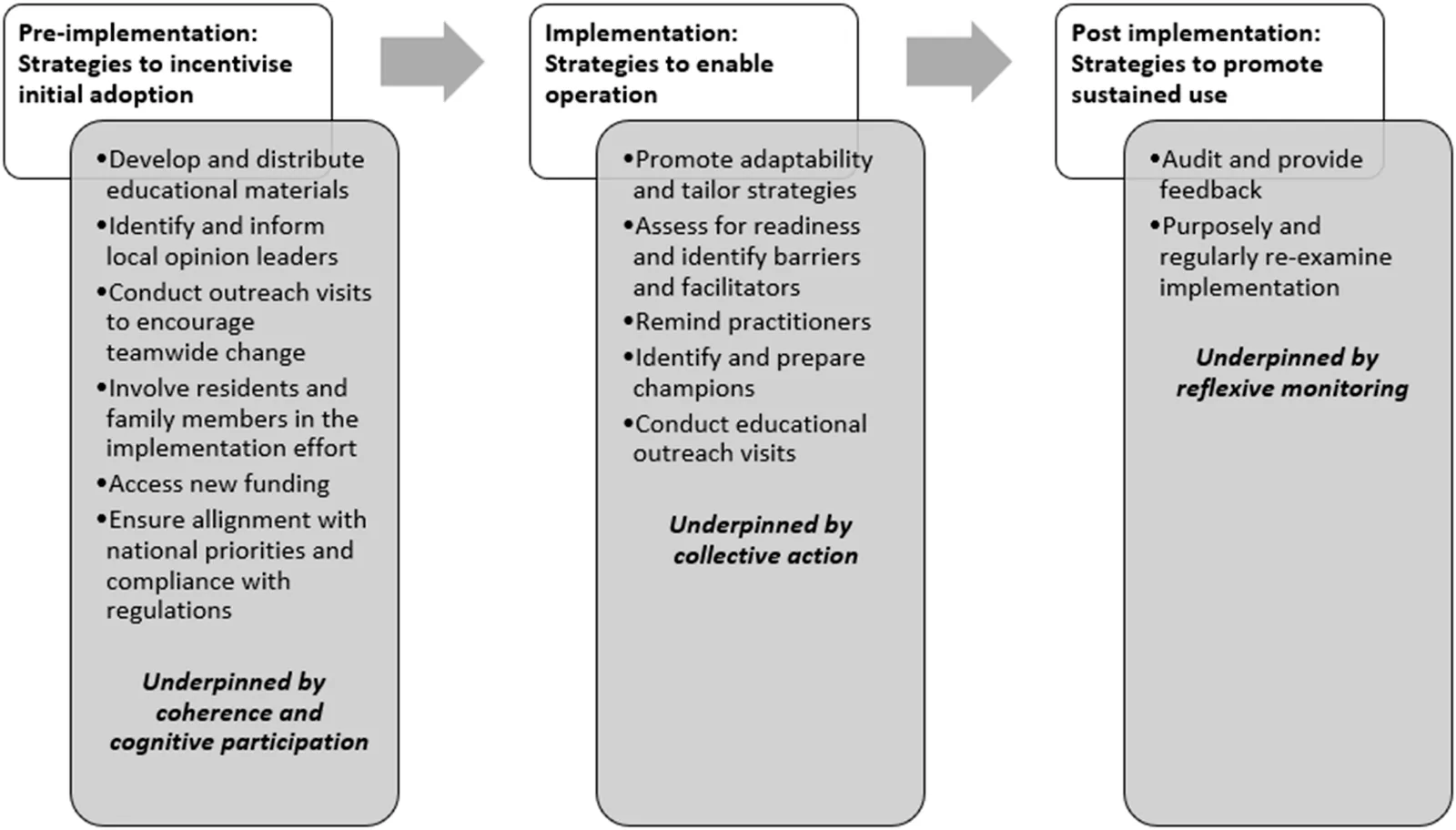
The implementation plan comprising three phases and corresponding implementation strategies, constructed from the synthesis of findings from the systematic review^25^ and co-design study.^26^

Throughout the co-design workshops, the four constructs of the NPT were collaboratively revised with participants into three sequential phases which formed the implementation plan, capturing and representing the key mechanisms for implementing the Framework in care homes. The findings indicated that for successful implementation, individuals must be (1) initially incentivised to adopt the Framework, relating to the coherence and cognitive participation constructs of the NPT; (2) able to practically operate the Framework, relating to collective action and (3) motivated to sustain its use, relating to reflexive monitoring. Each phase had its own implementation requirements and corresponding strategies (see Figure 1). Feasibility of the plan was tested in this study and reported in this paper.

### Measures and data collection

Exploration focused on a selection of five of Proctor’s eight implementation outcomes^28^ which allow for both assessment of the potential impact (adoption, fidelity and penetration) and inform ongoing development (acceptability and feasibility) of the implementation strategies (see Table 1).

**Table 1.** Implementation outcomes measured in this study.

Outcome purpose	Outcome measured	Definition	Outcome operationalised for the EMBED-Care framework	Data source (analysed in real time to generate iterative responses to barriers)
To assess the potential impact of the implementation strategies	Fidelity	Degree to which intervention can be implemented and adhered to as intended. Also refers to adaptations which were required to the original implementation plan in response to barriers	Measures of adherence - frequency of access as planned: - 1x minimum IPOS-Dem completed per resident per month Target: 80% of IPOS-Dems completed as intended (number of IPOS-Dems completed per month/expected number of IPOS-Dems if each registered resident were to receive it a minimum of once) as an indicator of fidelity and adherence. - Subsequent access of decision support tools and priorities of care list 2) Adaptations made to implementation strategies	- App user data - Observation - Interviews/focus groups
	Adoption at site and staff level	Decision or action to employ the intervention	*Site level:* number of care homes approached, reasons not for participating. *Staff level:* extent to which staff adopted the Framework. Target: 80% of recruited staff use the Framework (number of staff completing an IPOS-Dem/total number of recruited staff) as an indicator of adoption	- Field notes - App user data - Observation - Champion meetings
	Penetration	The extent to which an intervention is integrated within a setting	Penetration: extent to which the Framework was integrated into the care home: Target: 80% of registered residents receive the intervention (number of residents IPOS-Dem completed with/total number of residents registered on the app) as an indicator of penetration	- App user data - Observation
To inform development of the implementation strategies	Feasibility	Extent to which the intervention and implementation strategies can be successfully used	Usage measures of adherence: frequency, duration, and adherence as planned (see fidelity section above). Staff perceptions of whether implementing the Framework was feasible and a suitable fit, and what influenced use.	- Training evaluation form - Champion meetings - App user data - Observation - Interviews/focus groups
	Acceptability	Stakeholder’s perception that implementation is agreeable	Whether participants found implementation of the Framework agreeable in terms of content, delivery and complexity.	-Training evaluation form - Champion meetings - Observation - Interviews/focus groups

Data were collected for a period of 16 weeks, from December 2023-March 2024. Qualitative data comprised focus groups, semi-structured interviews, non-participant observations and field notes from champion meetings documenting contextual factors influencing engagement. Quantitative data included participant demographics, eHealth usage metrics, and training evaluation (see Table 2). As all data collection tools and study materials were developed by the research team, obtaining necessary permissions was not required; all the materials are freely available online.

**Table 2.** Data collection methods.

Data collection methods	
Qualitative data	
Champion meetings field notes	Champions were identified in each care home, with the intention that they would drive implementation of the Framework, support other staff and initiate its use to bring about a change in routine practice. Fortnightly contact was made with champions, exploring real-time user-experience of using the Framework, to inform iterative development of the implementation plan.
Focus groups and semi-structured interviews	Twelve weeks after study opening, focus groups were conducted with care home staff. Where they were unable to attend, a separate interview was conducted. The purpose of the focus groups was to collectively explore experience of use, perceptions of the Framework’s content and design, and discuss suggested modifications to implementation. Interview topic guides and focus group materials were developed using findings from the systematic review, output from the co-design workshops and the NPT. All topic guides were developed along side, and pilot tested by the EMBED-Care PPI group (See Appendix 2).
Non-participant observations	Structured non-participant observations of the Framework in use were conducted; these have previously been documented to provide insight into features of the implementation context which cannot be gained from interviews alone.^29^ Observer effects were mitigated by conducting naturalistic observations of the Framework; the schedule was dictated by when assessments were due, as indicated by notifications on the app, without researcher interference.
Training evaluation forms	The training evaluation form was largely qualitative, with free text questions asking participants to elaborate on what in the session was helpful or unhelpful, and on anything they would change. Participants were also asked to score whether “the face-to-face training and introduction was helpful and sufficient ahead of using the Framework” on a Likert scale (5=strongly agree, 0=strongly disagree).
Quantitative data	
Demographic data	Demographic data, including gender, ethnicity, job title, years of experience and years in post were collected for staff at baseline via a simple self-completed questionnaire. Aggregated, anonymised data on the characteristics of residents who received the intervention included gender, ethnicity, age and diagnosis.
eHealth usage data	Data pertaining to uptake (as an indicator of adoption and penetration) and adherence (as an indicator of feasibility of use and fidelity) were automatically collected electronically throughout the 16 weeks. A priori targets to indicate whether implementation had been successful were delineated ahead of site opening. A target of 80% was set for adoption, penetration, and fidelity in line with previous feasibility studies in similar contexts,^30,31^ with recommended progression criteria co-produced between patients, clinicians and researchers specifically for feasibility studies.^32^

### Data analysis

Descriptive statistics were conducted on quantitative eHealth usage data, and the training evaluation form data, to draw conclusions on implementation of the Framework. Qualitative data were synthesised and analysed using codebook thematic analysis, grounding the analysis in theory whilst allowing for the addition of new codes.^33^ The codebook comprised the three overarching phases of implementation which encapsulate the key requirements for implementing the Framework in care homes (see ‘Underpinning theory’ and Figure 1 above). Analysis was supported by NVivo 14.^34^

The lead researcher (JG) critically engaged in data familiarisation and initially deductively coded the data. The codes were subsequently revised and sorted into potential themes and sub-themes around the requirements for implementation at each phase. Where data did not align to a theme or sub-theme, an inductive approach was undertaken to elucidate further implementation requirements identified through real-world testing of the Framework in practice. Initial coding was conducted by one researcher (JG), with sense-checking and discussions at each subsequent stage of refinement by two others (CE and ND). Throughout analysis, the primary researcher drew on reflexive field notes to examine how her social and cultural position as a white female PhD fellow with a Psychology and mental health background shaped assumptions and supported data analysis. The Standards for Reporting Implementation Studies (StaRI) checklist was used to facilitate transparent and comprehensive reporting (see Appendix 1).^35^

### Data integration

Qualitative data sources were triangulated to enhance understanding, rigour, and credibility of the findings.^36^ They were complemented by, and integrated with, quantitative data around use and uptake of the Framework at the stage of data interpretation, to address the study aim, and provide a deeper understanding of how it was being implemented.^37^

Qualitative and quantitative findings were analysed independently initially, and synthesised through triangulation at the stage of data interpretation.^37^ Quantitative eHealth usage data provided an indication as to how the Framework was used. Qualitative data were used to explain what influenced the implementation process and provide richer insight into staff perspectives and experience. The findings informed the final iteration of the implementation plan.

## Results

### Characteristics of participating care homes and participants

Both care homes were residential and dementia-registered, situated in Greater London, and were privately owned. They were of a similar size, with 43 beds (care home one) and 39 beds (care home two); they had recently been rated by the Care Quality Commission (CQC) as ‘good’ and ‘outstanding’ respectively.^38^

### Care home staff

Twenty staff members were recruited across the two care homes, including the champions (n=5). Six focus groups (staff n=18) and one interview for a staff member unavailable to attend the focus groups, were conducted three months after the app was introduced in the care homes. Characteristics of participating staff are detailed in Table 3.

**Table 3.** Participating staff demographics.

​	​	Care home one (n=10)	Care home two (n=10)
Gender	Male	2	1
	Female	8	9
Ethnicity	Indian	3	4
	Other Asian	4	​
	Bangladeshi	​	1
	Filipino	1	​
	African	1	​
	White and Black African	1	1
	White British	​	4
Job title	Care assistant	2	3
	Senior carer	7	4
	Team leader	1	​
	Training and development manager	​	1
	Care co-ordinator	​	1
	Registered nurse	​	1
Number of years care experience	Less than 1 year	2	​
	1-4 years	6	3
	5-9 years	1	3
	10+ years	1	4
Number of years in current post	Less than 1 year	2	2
	1-4 years	6	4
	5-9 years	1	1
	10+ years	1	3

### Residents and family carers

Twenty-six residents received the intervention over the four-month period. Of these, the care home managers assessed that none had capacity to give consent to participate in research, and therefore no interviews with residents were conducted.

Non-identifiable data on the characteristics of residents who received the intervention over the implementation period were provided by the care home staff (see Table 4 for details).

**Table 4.** Characteristics of residents who received the intervention.

​	​	Care home one (n=12)	Care home two (n=14)
Gender	Male	1	3
	Female	11	11
Ethnicity	White British	12	13
	White Irish	​	1
Age	70-80	3	2
	81-90	7	8
	90+	2	4
Diagnosis	Alzheimer’s	3	5
	Vascular dementia	1	3
	Mixed dementia	3	1
	Cognitive impairment Other (inc. no formal diagnosis)	5	5

Twenty family members were approached but some declined participation due to prior commitments, concern over the study causing distress, health issues or other undisclosed reasons (n=8). Two family members were uncontactable despite three attempts (via email and telephone call). The remaining twelve family members were recruited into the EMBED-Care programme but outside the recruitment timeframe of this feasibility study.

### Key findings

#### Adoption at setting level: To what extent did eligible care homes adopt the intervention?

Thirty-two care homes were contacted via email or telephone from March 2022-May 2023, giving them details of the EMBED-Care study, with a view to asking them to participate. Of the 32, 18 did not respond after a second contact attempt. Of the 14 responders, two agreed to participate, however, due to issues with the app functionality at that time, and subsequent delays to the planned start date due to information governance both care homes ‘withdrew’ ahead of the feasibility study. Two care homes declined as they were unable to support delivery of the study, or it was unsuitable for their population. Five replied requesting more information, but on provision of additional detail, or a meeting request, did not respond further. Three others which had been contacted expressed an interest in participation, but after the recruitment target had been reached and recruitment had closed. The final two care homes went forward with the study.

#### Adoption at staff level: To what extent did eligible staff adopt the intervention?

Of 21 eligible staff, 20 consented to participate; one declined due to concerns around workload.

There was wide variation in the number of staff accessing the Framework. In both care homes, staff use was at its lowest in December and peaked in January, with 60% (n=6) and 90% (n=9) of staff using the Framework, when researcher presence was at its highest ahead of the focus groups. Detail can be found in Table 5.

**Table 5.** eHealth usage data summary for both care homes.

Care home	December		January		February		March	
	CH1	CH2	CH1	CH2	CH1	CH2	CH1	CH2
No. IPOS-Dems completed/total number of registered residents (%)	0% (0/0)	30% (3/10)	70% (7/10)	92% (12/13)	54.5% (6/11)	62% (8/13)	75% (9/12)	57% (8/14)
No. staff completed an IPOS-Dem/all staff registered (%)	0% (0/0)	30% (3/10)	60% (6/10)	90% (9/10)	30% (3/10)	60% (6/10)	10% (1/10)	60% (6/10)
Decision support tools accessed (n)	0	0	23	7	5	0	2	0
Instructional manual accessed (n)	0	1	4	1	0	0	0	1
Training videos accessed (n)	0	1	9	2	0	0	0	0
Priorities of care list completed (n)	0	1	4	0	0	0	0	0

#### Penetration: To what extent did registered individuals receive the intervention?

A final total of 26 residents were registered on the app, which cumulatively increased over the period of data collection. In both care homes, the number of residents who received the intervention was at its lowest in December and peaked in both care homes in January at 70% (n=7) and 92% (n=12) for care homes one and two respectively (see Table 5).

#### Fidelity: To what extent was the intervention implemented as planned?

Overall, the Framework was not implemented as intended. Across both care homes, with the exception of one month in care home two, the IPOS-Dem assessment was conducted with under 80% of residents who were registered on the app. Table 5 provides detail on which elements of the Framework were accessed, and how frequently, derived from the eHealth usage data. The decision support tools and training resources (manuals and training videos) were also accessed substantially less than anticipated.

#### Feasibility and acceptability: What factors influenced implementation of the Framework?

Findings influencing the Framework’s implementation were categorised into the three themes, corresponding to the three phases of the implementation (see Figure 1) underpinned by the constructs of the NPT: Requirements for 1) adoption, 2) operation and 3) sustainability of the Framework.

### Adoption: 1) What is required to incentivise adoption of the Framework prior to implementation?

#### Incentivising care home staff

Staff largely understood the purpose of the Framework and recognised its theoretical benefit to care delivery. These included its potential to enhance person-centred care and help staff *‘understand more about the residents’* (CH1, I1, care assistant), and its ability to improve timely assessment of symptoms ‘*to help support the person in terms of likely additional care for their needs*’ (CH2, FG2, senior carer). They appreciated that the Framework could provide *‘clear information’* (CH1, FG1, care assistant), and empower staff to be more *‘self-sufficient’* in make appropriate decisions without external input.

The positive impact of the Framework on staff’s personal development and career progression was also recognised, with staff acknowledging ‘*it’s a good thing for my future’* (CH02, FG3, care assistant), echoing sentiments voiced when co-designing the implementation plan.

#### Barriers to incentivisation

The potential advantages of utilising the Framework were evidently not realised in practice. The perception that the Framework was ‘*a good experience, but…didn’t benefit’* staff and residents (CH2, FG1, champion), was salient in both care homes and diminished motivation to engage with it; merely appreciating the Framework’s potential was insufficient to translate into initiation of use.

Additional barriers to adoption included staff concerns about the use of technology in this context, and that it could de-personalise care delivery and interfere with the patient-practitioner relationship. Staff were concerned about generational differences in attitudes towards technology - ‘*the residents think that we are doing something on our phone so ignoring them…that’s extremely rude*’ (CH2, FG1, team leader).

Unfamiliarity with technology was echoed in some of the care staff’s own apprehensions around adopting the Framework - ‘*me myself are not really young* [laughs], *my brain is overload!’* (CH1, FG1, senior carer). Ensuring that additional support is provided, to accommodate different technology competencies, will be vital if eHealth is to be feasibly adopted by all staff.

#### Family involvement

When co-designing the Framework, insufficient opportunity for families to be involved was proposed as a key barrier to adoption; working with families is inherent to the definition of good person-centred care. Despite encouragement from the research team, staff didn’t seek to include relatives in practice, with concerns about how the Framework *‘would cause distress’* as ‘*some families don’t understand dementia… (the notifications) would cause alarm… straightaway think they think death’* (CH02, FG1, champion).

### Operation: 2) What is required to operate the Framework?

#### Compatibility

Operation of the Framework was impeded by a consensus amongst staff that the assessing resident symptoms via the app was ‘*exactly the same really, no different’* (CH2, FG2, senior carer) to those which they were already routinely performing, and any unmet needs would have already been detected at the point of care. There was a sense the app was superfluous, with it only identifying ‘*all the things we are already monitoring everyday’* (CH2, FG2, senior carer).

Whether perceived duplication of processes was specific to the individual care home was raised. Both care homes had high CQC ratings and reported good integrated working with external healthcare professionals, with ‘*brilliant rapport with the GP, the nurses, everybody’* (CH02, FG1, senior carer) and immediate access to input when required. Staff expressed that they were *‘all trained in everything…and already ahead in our everyday way’* (CH02, FG2, champion), suggesting that the Framework might be found more useful in the many homes which are less well resourced, or *‘not as passionate about training as* [manager name] *is, or as we are and they would benefit’* (CH1, FG1, carer).

There were also concerns around the Framework’s length; staff found that the Framework was unrealistic to access, digest and action the Framework on shift, *‘Eight or twelve [decision support tools] there? It will be sunset and I haven’t served tea’* (CH1, FG3, senior carer). Questions about the necessity, utility and practicality of incorporating the Framework into their workflow inhibited uptake.

#### Workforce challenges

Across both care homes staff reported that, even before introducing the Framework, they were at full capacity. This was reported by senior care staff undertaking additional roles due to a reduced workforce, ‘*whenever there is no assistant manager we will be taking over her role. our time is really very limited’* (CH1, FG3, senior carer). Observations revealed that use of the Framework was often interrupted by a call for medication or mealtimes.

Staff also cited the care home manager as an obstacle to using the Framework, reporting that ‘[care home manager] *doesn’t really want to take people off the floor’* (CH2, FG2, senior carer). Some had to find time outside of their shifts to access it, *‘I’m on a day off, I’m spending time here to do the training’* (CH2, FG3, care assistant). Understandably, this was perceived as unacceptable and caused discontent amongst staff members; justifiable frustrations impeded willingness to co-operate, and therefore the uptake of the Framework.

#### Staff support

Strategies in place to ensure that staff had adequate support to implement the Framework included a three-hour face-to-face training session, and inbuilt training videos and resources on the app.

Eighty six percent ‘strongly agreed’ that the face-to-face training/introduction was helpful and sufficient ahead of using the Framework. Mixed feedback was later received at the focus group. Some staff reported that they ‘*had enough training, and the support is there’* (CH2, FG1, senior carer), whereas others requested further training, saying ‘*what it literally boils down to, is the lack of knowledge of what we’re using’.* (CH2, FG2, senior carer). Ensuring that the amount of support, and training content, is tailored to the individual staff member’s needs, competency and ability is vital for successful uptake across the team.

There was similarly mixed feedback concerning the role of the champions. Staff generally appreciated their support and expressed a preference to receive training from the champions rather than the research team, reporting it was ‘*more helpful’* CH2, FG3, care assistant). However, the champions were largely senior staff members and were themselves overstretched. They were often not available, with staff reporting that *‘we have to go to them [the champions] just to understand what’s going on, and then they’re not here'* (CH2, FG2, senior carer). All staff were offered additional training to circumvent this, but the challenge of them taking time away from the care home floor impeded this.

It became quickly evident that when the research team were not present, the app was barely being used. This was confirmed in the user data; app use peaked in both care homes in January in accord with heavy researcher presence to encourage staff use ahead of the focus groups.

#### Appropriate digital infrastructure

Two tablets were provided in each home. Limited staff capacity coupled with malfunctioning technology understandably led to annoyance, and obstructed further use. Wi-Fi issues exacerbated frustrations with the app, with the *‘internet not working sometimes’*, and being *‘so slow’* when it did (CH2, FG3, care assistant).

Interoperability of the app with electronic devices already in use was explored. This was an agreeable potential improvement for staff, who agreed it would be *‘more convenient and accessible’* (CH1, FG2, senior carer), and that being able to access the app on their usual devices would save time as they were ‘*always on hand… on every floor’* (CH1, FG1, senior carer).

#### Reminders for use

Electronic alerts were built into the app to notify users when an assessment was due, but were only accessible once the app had been logged into. This was insufficient, with staff reporting the Framework was *‘not being part of routine’* leaving them *‘prone to forget it’* (CH1, FG1, senior carer) and citing this as a remediable barrier to its use. Additional reminders to instigate use of the app were requested when a task was outstanding, via email. The app developers informed us that this feature was not possible, so it was agreed the research team would manually send a monthly reminder to check the app for notifications, and that a delegated staff member would ‘*be a reminder, like an alarm clock to everyone”* (CH1, FG3, champion).

### Sustainability: What is required to sustain use of the Framework?

#### Feedback for users

Staff were able to access graphs illustrating a change in a resident’s symptoms over time via the web-based platform, providing a visual representation of any impact the Framework was having. While staff appreciated the possible value of this, to *‘see some improvement or not’* or ‘*see if they’re declining’* (CH2, FG2, care assistant), in practice, they were not utilising the graph function. This is of particular importance as the IPOS-Dem is an outcome measure to monitor change over time – underuse of this feature likely contributed to the sense that the IPOS-Dem duplicated current assessment tools.

#### Monitoring of implementation

Implementation strategies were iteratively revised in response to barriers to use over the period of data collection, aiming to promote sustained use. These revisions have been presented throughout the results section, are reviewed and summarised in the discission below. They are incorporated into the third and final iteration of the implementation plan which can be found in Appendix 3 (see column entitled ‘Adaptations and lessons learned following feasibility testing’).

## Discussion

In summary, although there was some evidence of uptake, eHealth usage data indicated that the Framework was largely not utilised as intended, and feasibility of the implementation strategies did not guarantee uptake of the Framework. Except for staff adoption in care home two one month after the study opened, none of the implementation a priori outcome targets were reached. The focus group data corroborated this; initial enthusiasm around enhancing provision of dementia care did not translate into sustained time investment when staff were faced with workforce challenges, concerns around compatibility and duplication of processes, and uncertainty around any additional benefit to care processes and resident outcomes.

### Compatibility with the care home context

Considering the nuances of the individual context in which an eHealth innovation is to be implemented is vital. Much implementation science research has been conducted in health care settings; care homes, as part of the social care system, pose a unique set of implementation challenges are requirements.

Compatibility with routine practice is a well-documented challenge in the implementation literature for long-term care settings.^39^ Overcoming this requires a thorough pre-implementation assessment of the specific context, to identify how the Framework might feasibly fit alongside, and what elements need be adapted, to promote readiness for change. That the pre-implementation phase is a ‘critical period’ in the process is increasingly recognised.^40^

Engagement with the Framework was diminished by a perception that it was duplicating the assessment processes already in place; it was being used as a questionnaire rather than as a PCOM to monitor changes in symptoms. Despite encouragement, staff were not accessing the web-based functionality to observe the impact of the Framework on residents’ symptoms over time, likely attributable to limited time and access to a desktop computer. Receiving feedback is understood as a key aspect of why individuals sustain use of outcome measures in routine care^41^; in future, regular automatic reports should be generated for staff, as observed in the successful implementation of PCOMs in palliative care settings.^42^

### Transitioning to eHealth

#### eHealth interoperability

Consistently observed in the literature, eHealth which cannot ‘communicate with’ other information systems or technology is a key barrier to use.^43^ A lack of appropriate digital infrastructure, including issues with Wi-Fi paired with inability for the Framework to be accessed offline, impeded uptake: another commonly encountered obstacle to eHealth implementation in care homes.^44^

Interoperability between systems is essential to promote the integration between care homes as part of social care and primary and secondary health care services, and to enable the secure transmission of sensitive resident-identifiable data.^43^ One of the requirements of the commitment to Enhanced Health in Care Homes (EHCH) laid out in the NHS Long Term Plan in the UK was that by 2024, every care home would have established protocols for information sharing, shared care planning and use of shared care records with their primary care network (PCN). How far this has been achieved is unclear; whilst it signalled a new direction for care delivery it is certainly not complete. Ensuring that any eHealth innovations are interoperable with the centralised communication system will be imperative if they are to be adopted in care homes.

#### The impact of eHealth on practitioner-resident interaction

One further barrier to transitioning to eHealth is a concern around ‘dehumanising’ care delivery. Staff were apprehensive about residents perceiving them as ‘rude and distracted’ if they were using an electronic device to conduct an assessment, de-personalising their traditional notions of receiving personal face-to-face care. The negative impact of eHealth on the practitioner-patient interaction in this way is well documented,^45^ and is of particular concern with this population who may lack cognisance that the device is ultimately being used to enhance person-centred care provision. Including individuals with whom eHealth will be used during its design and development, so that they are comfortable with its nature and purpose, is essential to mitigate these feelings, by ensuring it is intuitive, person-centred and responsive to their needs.^46^

### Staff workload and support

There was an evident mismatch between carers’ capacity to enact the Framework, and their ability and willingness to use it. Staff felt unable to allocate time in their day to complete their assessments; this is consistent with published findings, which have identified ‘organisational slack’ - or lack of it – as amongst the biggest influences on the uptake of innovation in the care home setting.^47^

#### Tailoring staff training

There was wide variation in the amount of training and support staff members required, depending on their individual aptitudes and confidence; this presents a serious challenge for designing an appropriate general training package. Care home staff are a diverse group^13,48^ with a huge range of previous experience; some are school leavers, while others are qualified clinical professionals in their country of origin but are often, wastefully, unable to register or practice despite extensive skills and experience.

There is also huge variation amongst working adults in the UK with regards to e-literacy and familiarity with technology.^43^ These differences need to be considered more carefully in future implementation of the Framework, recognising the need for individualised and adaptable training.

#### Champions and leadership engagement

Although champions sometimes provided necessary support to other staff, they too had limited time to offer in practice. Ensuring that expectations of the ‘champion’ role are clearly understood is important to ensure that the delegated individuals can fulfil their responsibilities; a finding highlighted elsewhere.^49,50^

Lack of senior staff engagement, and a consequently unconducive learning environment, are well-established barriers to uptake of new systems.^12^ Despite initial enthusiasm from one manager, it transpired that they were asking staff to attend training and other research related activities on their ‘days off’ to complete study tasks, to avoid it interfering with their other duties. This negatively impacted staff’s perception of the research, provoking resentment and compromising willingness to participate. Although securing the commitment of influential opinion leaders was a key strategy in co-designing the implementation plan, participants advocated more strongly for a ‘bottom-up’ approach, led by those who delivered direct care to residents. This echoes findings in other studies, where successful implementation of psychosocial interventions to improve dementia care in the residential setting required active engagement of the whole team.^51^

### Strengthening the implementation plan

The findings from this study have culminated in revised and strengthened strategies to guide the implementation of eHealth for use in care homes for people with dementia, using the Framework as a test intervention. The third and final version of the implementation plan can be found in Appendix 3.

Building on the findings from this feasibility evaluation, seven core elements were identified as fundamental to implementing eHealth interventions in care homes. These have been translated into as a set of practical recommendations applicable across all three phases of implementation. Each recommendation reflects learning from the EMBED-Care feasibility study and is underpinned by the constructs of the Normalisation Process Theory (NPT) - coherence, cognitive participation, collective action and reflexive monitoring - which together explain how interventions become embedded and sustained in practice. Table 6 summarises these recommendations, their theoretical basis, and illustrative insights from the feasibility evaluation.

**Table 6.** Recommendations for implementing eHealth in care homes: Core elements and theoretical underpinnings from the EMBED-Care feasibility study.

Core element	Recommendation for implementation	Illustrative learning from the feasibility study	Linked NPT construct(s)
1. Understand and assess local context	Undertake a structured assessment of each care home’s organisational context, workforce capacity, digital infrastructure, and readiness for change before implementation. Use findings to tailor timelines, training and support.	Readiness assessments revealed key barriers—limited Wi-Fi connectivity, workload pressures and duplication of assessments. Tailoring strategies to context improved engagement and feasibility.	*Coherence* (understanding purpose and value); *Collective action* (aligning work with local systems).
2. Engage end-users and families from the outset	Involve care staff, managers, residents (where appropriate) and family carers early in planning and design to build ownership, legitimacy, and shared understanding of purpose.	Staff engagement through co-design generated enthusiasm, but family participation remained limited in practice. Structured opportunities (e.g. family meetings, coffee mornings) are needed for inclusive implementation.	*Cognitive participation* (enrolling and engaging others); *Coherence*.
3. Provide flexible and ongoing staff support	Offer adaptive, continuous support that recognises variation in staff roles, experience and digital literacy. Combine modular and refresher training, peer support, and easily accessible troubleshooting.	Staff valued initial training but required continued guidance. Champions and researcher presence were crucial for sustaining engagement. Ongoing internal support structures are needed post-implementation.	*Collective action* (enabling people to enact the intervention); *Cognitive participation*.
4. Integrate eHealth tools with existing workflows	Align eHealth systems with routine care processes to prevent duplication. Map workflows in advance and identify where the tool complements current assessments.	Staff perceived the Framework as replicating existing processes. Clarifying its added value as a person-centred outcome measure and embedding results in routine reviews can enhance perceived usefulness.	*Coherence* (sense-making); *Collective action*.
5. Strengthen digital infrastructure and interoperability	Ensure stable connectivity, appropriate devices, and compatibility with existing electronic care records prior to roll-out. Provide contingency plans (e.g. offline functionality).	Technical issues (slow Wi-Fi, non-functioning tablets) were major barriers. Integrating the app with existing digital systems and enabling offline access were identified as key requirements.	*Collective action* (resource adequacy and integration of effort).
6. Promote adaptability and real-time learning	Treat implementation as cyclical and iterative. Establish mechanisms for ongoing reflection, feedback and strategy adaptation in response to emerging challenges.	Regular champion meetings and usage-data reviews facilitated responsive adjustments (e.g. tailored training, reminders). Real-time adaptation promoted learning and sustained engagement.	*Reflexive monitoring* (appraising and adjusting); *Cognitive participation*.
7. Refine the intervention to fit user needs	Co-design refinements with end-users to improve usability and relevance. Simplify interface and content, and generate concise, actionable outputs to support decision-making.	Staff found the Framework lengthy and dense. Streamlining content and introducing automatic summaries or AI-supported recommendations could enhance fit within care-home routines.	*Reflexive monitoring* (evaluating effects); *Coherence*.

Previously, the three phases of implementation were considered as linear and distinct: the NPT is often regarded as a temporal process,^52^ and in this study, it was supposed that the mechanisms of change required to implement the Framework would operate sequentially. However, real-life testing of the implementation plan demonstrated that the requirements at each phase are, in practice, interlinked and cyclical.

**Figure 2.**
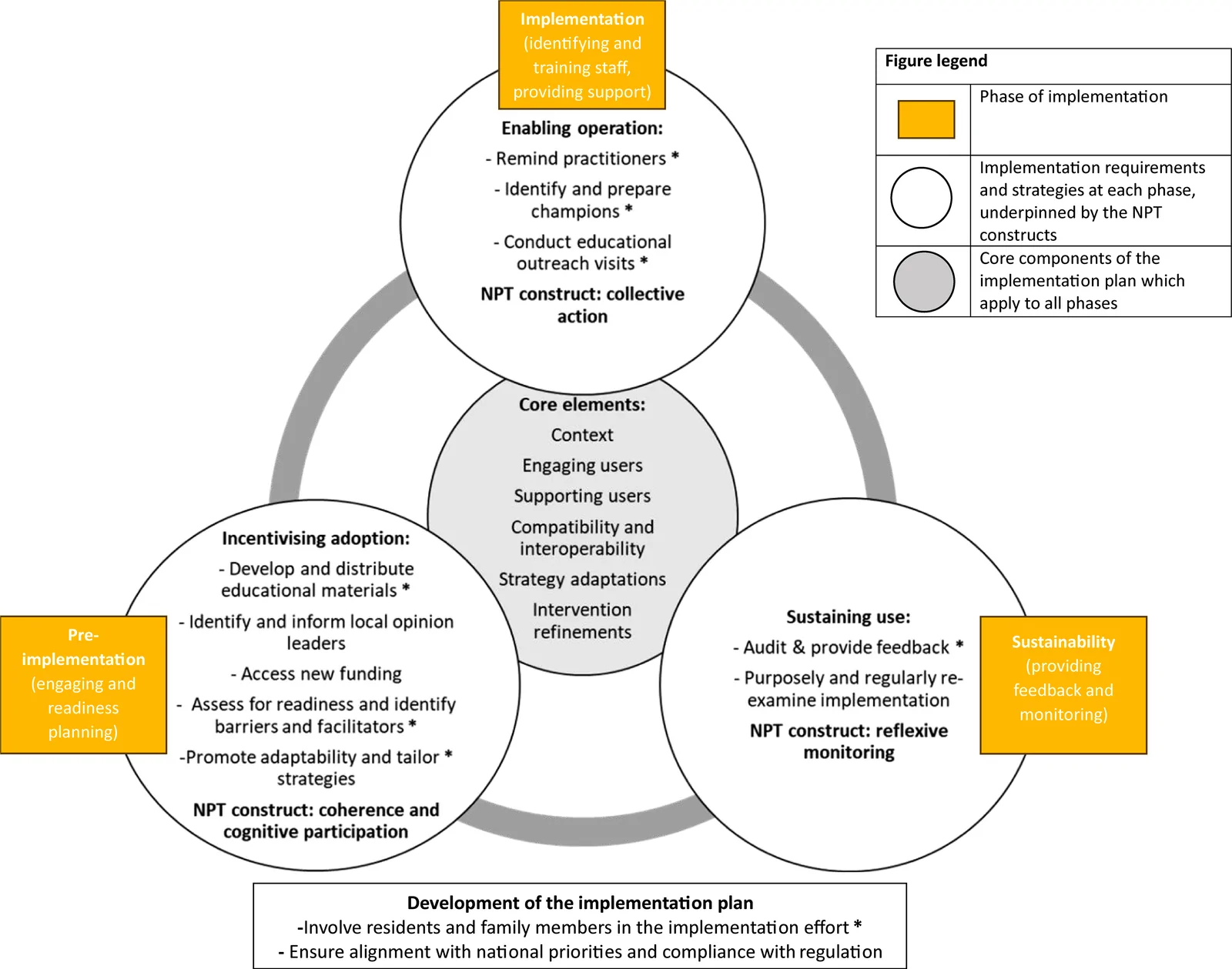
An overview of the implementation plan incorporating findings from the feasibility study.

This has implications for the NPT constructs which underpin the phases of implementation, echoing published findings reporting simultaneous experience of the NPT constructs,^53^ and a dynamic interaction between them.^54^ Figure 2 depicts this revision to the implementation plan. Whilst there are still overarching phases with related requirements and strategies, these are more cyclical and iterative than anticipated. The phases occur simultaneously and might be revisited severally throughout one implementation project. The revised strategies are marked with an asterisk in Figure 2, and the revisions explained in detail in the final iteration of the implementation plan in Appendix 3.

### Implications for the intervention

A key barrier to implementation related to the design of the intervention; amendments to the plan will be little effective without first strengthening the ‘fit’ of the Framework with the requirements of the care home context.^55^

Although the IPOS-Dem was considered by some to be too lengthy, it is more than an assessment tool; its merit lies in its comprehensiveness, and ability to identify and monitor symptoms spanning multiple health domains over time.^56^ Use of PCOMs in palliative care have been shown to improve communication between health and social care practitioners, inform decision about priorities for care and evaluate patient outcomes^57^ - critical in residents with complex and varied needs.

The intention was that the app would automatically combine the relevant support tools for each respective IPOS-Dem item, but in practice this linkage was not possible, and the content presented was excessive to digest at the point of care. A streamlined synopsis of the support tools is required, tailored to the results of the IPOS-Dem, in which the key points are collated into one output. Integrating Artificial Intelligence (AI) as a step in-between completion of the IPOS-Dem and presentation of the decision support tools could allow for quick collation of all the relevant information into a succinct tailored output. The potential of AI to enhance dementia care is becoming increasingly recognised^58^; the possibility and suitability of incorporating it into the Framework will be the subject of future enquiry.

### Strengths and limitations

The implementation strategies presented here are rigorously underpinned by theory and have been subject to testing in practice. Originally co-designed with people affected by dementia, they have been refined in response to barriers identified by individuals trialling them in the real-world care home context. The implementation plan has been strengthened by collaborating with users prospectively in its design and in subsequent evaluation. It is context specific and can be used to guide implementation of other eHealth interventions for people with dementia in residential care homes. Making iterative adaptations to the plan in real-time informed learning and allowed for the strategies to be responsive and flexible. Unfortunately, there was insufficient time to re-evaluate the plan following all the changes made. Future research will explore whether these amendments were effective.

A key finding from systematic review,^25^ was the importance of considering residents’ person-centred needs and involving end-users in the implementation effort. Originally, the intention was that both residents and family carers would partake in focus groups and interviews to understand their experiences of using the Framework. Unfortunately, none of the residents in the study had capacity to participate, and it was not possible to recruit any family members within the study timeline. A potential limitation relates to the reliance on care home managers as gatekeepers in determining residents’ capacity to participate. While this approach was necessary for ethical and practical reasons, it is possible that assumptions were made which may have excluded individuals who could have meaningfully contributed, thereby limiting the inclusivity and representativeness of the sample.

Resident and family involvement is integral to good person-centred palliative care. Understanding family carer experience is crucial to minimise disconnect between families and staff, and promote more trusting and reciprocal relationships which facilitate shared decision-making. Future enquiry will involve implementing the Framework with residents in the earlier stages of dementia and working more closely with family members to encourage increased participation.

## Conclusion

Normalising use of an eHealth innovation for a population with complex care needs into a context facing pre-existing systemic issues engendered a series of challenges. Evaluating the feasibility of the implementation plan on a small-scale was a valuable and important step in its development; rapid evaluation, and iterative response to barriers to use, informed learning and allowed for real-time adjustments to strategies. These largely related to providing further support for staff and promoting engagement; however, these will be ineffective without simplifying the Framework to suit the care home context. Theoretically informed and tested in practice, the findings have been incorporated into the final iteration of the implementation plan. The next step is wider involvement of family members and residents in care homes to refine implementation strategies ahead of further large-scale testing, to inform future research and wider use of eHealth interventions in practice.

## Supplemental material

Supplemental material - Implementing the empowering better end of life dementia-care (EMBED-care) framework to improve palliative dementia care in care homes: A feasibility evaluation Supplemental material for Implementing the empowering better end of life dementia-care (EMBED-care) framework to improve palliative dementia care in care homes: A feasibility evaluation by Juliet Gillam, Nathan Davies, Clare Ellis-Smith, Ayesha Dar, Elizabeth Sampson, Catherine J. Evans on behalf of the EMBED-Care programme in Journal of DIGITAL HEALTH

Supplemental material - Implementing the empowering better end of life dementia-care (EMBED-care) framework to improve palliative dementia care in care homes: A feasibility evaluation Supplemental material for Implementing the empowering better end of life dementia-care (EMBED-care) framework to improve palliative dementia care in care homes: A feasibility evaluation by Juliet Gillam, Nathan Davies, Clare Ellis-Smith, Ayesha Dar, Elizabeth Sampson, Catherine J. Evans on behalf of the EMBED-Care programme in Journal of DIGITAL HEALTH

Supplemental material - Implementing the empowering better end of life dementia-care (EMBED-care) framework to improve palliative dementia care in care homes: A feasibility evaluation Supplemental material for Implementing the empowering better end of life dementia-care (EMBED-care) framework to improve palliative dementia care in care homes: A feasibility evaluation by Juliet Gillam, Nathan Davies, Clare Ellis-Smith, Ayesha Dar, Elizabeth Sampson, Catherine J. Evans on behalf of the EMBED-Care programme in Journal of DIGITAL HEALTH

## Data Availability

The datasets used and/or analysed during the current study are available from the corresponding author on reasonable request.

## Appendix

### Abbreviations

CFIR: Consolidated Framework for Implementation Research
CQC: Care Quality Commission
CRN: Clinical Research Network
EHCH: Enhanced Health in Care Homes
ERIC: Expert Recommendations for Implementing Change
EMBED-Care: Empowering Better End of Life Dementia-Care
FRAME-IS: Framework for Reporting Adaptations and Modifications to Evidence-Based Implementation Strategies
GP: General Practitioner
HRA: Health Research Authority
IPOS-Dem: Integrated Palliative care Outcome Scale for Dementia
NHS: National Health Service
NPT: Normalisation Process Theory
PCN: Primary Care Network
PCOM: Person-Centred Outcome Measure
PPI: Patient and Public Involvement
REC: Research Ethics Committee
StaRI: Standards for Reporting Implementation Studies.
