# Supplementary material for: Implementing the Empowering Better End of life Dementia Care (EMBED-Care) Framework to improve palliative dementia care in care homes: A feasibility evaluation
Source: Digit Health. 2026 Sep 8;12:20552076261476137. doi: 10.1177/20552076261476137 (PMC13554648; doi:10.1177/20552076261476137)
Supplement: Supplementary file 2 [file sj-pdf-2-dhj-10.1177_20552076261476137.pdf]

Focus group guidelines and questions: care home staff

Thank you for agreeing to take part in this discussion group. The purpose of the discussion is to understand your experience of using the EMBED-Care Framework with the residents participating in the study.

In particular, we would like to get your perspective on whether the EMBED-Care Framework plays a role in supporting the care you provide, whether there are any problems or challenges to using the EMBED-Care Framework, and whether you think any changes are required to help using the EMBED-Care Framework. Your participation is entirely voluntary (you can withdraw at any time) and you do not have to answer questions that you do not feel comfortable with.

- Recording
- Confidentiality
- Timing
- Feedback and suggestions

Introduction of attendees (name and role)

1)	5)
2)	6)
3)	7)
4)	8)

Interview/Focus Group Questions	Prompts
**General questions around use of EMBED-Care**	
**1. Can you describe how you have introduced and used the EMBED-Care Framework in the care home?**	Which staff are using the app (junior/senior/key workers)? How was the work allocated? Are they supported? How often is it used? How long does the assessment/decision making take? Does it vary? Which part takes the longest?
**2. How have you found using the app/technology to support a) assessment and b) decision making?**	Is it user-friendly e.g. language, lay out? Any tech issues? Duplication of work? Additional resources e.g. videos, manuals helpful/accessed? Alert system – helpful?
**3. Practical app considerations:**	Which devices are used (tablet, personal phone, desktop)? Interoperability with current record systems/care plans/apps in use?
**Causal mechanisms and potential benefit of using the EMBED-Care Framework**	
**1. Could you share whether/not the information gained from EMBED-Care Framework can be used to inform the care that you provide?**	Could you explain further?
**2. Did using the Framework impact assessment/identification of concerns?**	How or why/not? Did it improve identification of needs/concerns? One off? Ongoing monitoring? Duplicate or complement current assessments?
**3. If a concern was identified, did using the Framework impact decision making around care?**	Did anything change in how you make decisions? If not, why/what got in the way? Did it help prioritize/address concerns?

	If the app notifies staff of a red alert, what happens? Who is responsible for acting on it? How much time did it take to resolve?
**4. Did using the Framework impact communication about residents?**	Between staff, with other health care professionals, family members?
**5. Are you aware of any impact that using the Framework has had on your work/the residents?**	How do you know?<br>In what way, if any, have people's understanding of the value of EMBED-Care Framework impacted its use?
**6. Were there any benefits of using the EMBED-Care Framework?**	Has it been valuable? If not, why not?<br>Residents e.g., monitoring concerns, improving care delivery.<br>Staff e.g. workload, communication, confidence in role, escalating concerns.
**7. Were there any potential challenges too/harms of using the EMBED-Care Framework?**	To yourselves, to residents?<br>Challenges e.g. integrating the app into current record keeping processes, lack of staff time, tech problems, insufficient training, insufficient support, too complex, not understanding what's required of staff.
**8. How could these be overcome?**	Is there anything we could put in place to minimize/avoid these happening?
**Implementation**	
**1. Did anything encourage you to use the EMBED-Care Framework? If so, what?**<br>a) **IPOS-Dem**<br>b) **Decision support tools**	E.g., the aim of the Framework, less paperwork, workload benefits, desire to improve to care, personal development.
**2. Did you feel you supported to use the Framework? If so, how? If not, what might have helped?**	E.g. training – resources, face to face?, Champions, provision of tablets, researcher presence/contact, leadership encouragement. Did they impact adoption/use?
**3. Do you feel like the EMBED-Care intervention has a clear and shared aim and purpose?**	Does it make a clear difference?
**4. Does the Framework fit alongside routine assessment/decision making procedures in the home? If so, could you detail? If not, what might help this?**	Is there any duplication of work?<br>How is it different to your normal practice?
**5. What changes could we make/ factors might affect your decision to continue using the Framework?**	Changes e.g., additional app features, to videos/training, more support, more tablets, increased family carer access, interoperability with other EHR's, tailoring of notifications, changes to app layout/format.
**6. How have the team organized themselves to work together to use the Framework?**	Do you see using the Framework as part of your role?<br>In what way, if any, has support from colleagues/ management impacted use?
