# Supplementary material for: Implementing the Empowering Better End of life Dementia Care (EMBED-Care) Framework to improve palliative dementia care in care homes: A feasibility evaluation
Source: Digit Health. 2026 Sep 8;12:20552076261476137. doi: 10.1177/20552076261476137 (PMC13554648; doi:10.1177/20552076261476137)
Supplement: Supplementary file 3 [file sj-pdf-3-dhj-10.1177_20552076261476137.pdf]

Third and final version of the implementation plan incorporating findings from the feasibility study, reported in line with Proctor's guidelines for specifying and reporting implementation strategies.

Implementation phases	Implementation strategy	Adaptations and lessons learned following feasibility testing	Operationalised for the EMBED-Care Framework, reported as recommended by Proctor et al, 2013 (28)
**Development of the implementation plan**	*Involve residents and family members in the implementation effort*		**Actor(s):** Intervention developers, users, stakeholders, residents, and family carers<br><br>**Action:** Active involvement of key stakeholders in the co-development and evaluation of the implementation plan. Where appropriate, particular emphasis should be placed on involving residents and family carers in delivering the intervention.<br><br>**Target of the action:** To ensure the implementation strategies are acceptable, feasible and responsive to their users, and to promote equal partnership and shared decision-making.<br><br>**Temporality:** Prospectively, from the start of the study and development of the implementation plan, through to the testing in practice.<br><br>**Dose:** Once monthly co-design meetings to allow for iterative developments to be made to the plan in-between meetings. Interviews with residents and family members 12 weeks following implementation to understand and learn from their experiences.<br><br>**Implementation outcome affected:** Acceptability and feasibility of the implementation strategies<br><br>**Justification:** NPT (coherence and cognitive participation), CFIR (Implementation process: Engaging end-users, (Intervention

			characteristics: Evidence Strength and Quality and Intervention Source), systematic review, co-design workshop and feasibility study data analysis.
	Ensure alignment with regional and national priorities and compliance with eHealth regulations		**Actor:** Intervention developers<br><br>**Action:** To ensure the intervention aligns with requirements and recommendations for best practice and is compliant with regulations e.g. NHS Enhanced Health in Care Homes (EHCH) model and Gold Standards Framework, and to promote this in communications with the sites<br><br>**Target of the action:** Intention to adopt the intervention and promote the delivery of optimal dementia care aligned with evidence-based practice<br><br>**Temporality:** Prospectively throughout the development of the eHealth intervention and implementation plan<br><br>**Dose:** At all intervention and implementation plan development meetings, and promoted at meetings with potential sites<br><br>**Implementation outcome affected:** Adoption and acceptability of implementing the intervention<br><br>**Justification**: NPT (cognitive participation, coherence), CFIR (Intervention characteristics: Evidence Strength and Quality), systematic review and co-design workshops.
**Pre-implementation**<br><br>**Strategies to**	*Develop and distribute educational*	- Emphasising the unique added value of a PCOM to regular assessment procedures to enhance holistic person-centred care	**Actor(s):** Intervention developers<br><br>**Action**: Develop and distribute educational and instructional materials about the intervention to potential sites and family members via multiple

**incentivise individuals to adopt the intervention**<br><br>**Targets NPT constructs:** Coherence and Cognitive Participation	*materials*	(including examples in practice) is required to minimise perception of duplicating work.<br><br>**Adaptation**: When developing educational materials, make explicit that the Framework is more than just an assessment, the benefits of monitoring outcomes over time, and the importance of the linkage between the IPOS-Dem, decision support tools and priorities of care list for an improvement in evidence-based care delivery at the point of care. Ensure these are distributed to family members and residents.	platforms e.g. posters, flyers, twitter posts, presentations<br><br>**Target of the action:** To provide potential users with knowledge about the intervention, skills to use it and highlight its benefits to facilitate incentive for adoption. Promoting person-centred care through encouraging family involvement.<br><br>**Temporality:** Prior to implementation, following identification of sites<br><br>**Dose:** Each site should receive minimum one face-to-face presentation introducing the study, flyers, a poster for the care home and training materials following identification, made available to residents, staff and family members.<br><br>**Implementation outcome effected:** Adoption at staff and family level and reach to residents within the care home<br><br>**Justification:** NPT (promoting coherence and cognitive participation), CFIR (Individual characteristics: knowledge and beliefs), systematic review, co-design workshop and feasibility study data analysis.
	Identify and inform local opinion leaders and influencers	- Ensure the research recruitment process is conducted sufficiently in advance of planned study opening to recruit sites, but not so far in advance that circumstances change and sites withdraw or lose momentum.	**Actor(s):** Intervention developers, opinion leaders<br><br>**Action:** Schedule meetings with influential leaders e.g. Clinical Research Network (CRN) leads, ENRICH (Enabling Research in Care Homes) Research Ready Network, to promote the intervention and plans for implementation e.g. eligibility criteria, reimbursement plan, data collection plans. The opinion leaders will in turn identify and contact eligible sites and relay this information.<br><br>**Target of the action:** Care home managers of eligible sites are provided

			with sufficient information concerning the study so that they are engaged and incentivised to participate.<br><br>**Temporality:** At least 6m prior to planned study opening.<br><br>**Dose:** One face-to-face meeting with each team e.g. CRN lead for a specific area, with follow up meetings held if required.<br><br>**Implementation outcome affected:** Adoption at site level<br><br>**Justification:** NPT (cognitive participation), CFIR (Inner setting: leadership engagement), systematic review, co-design workshop and feasibility study data analysis.
	Conduct educational outreach visits to encourage teamwide change	- Although encouraged ahead of implementation, family members had little involvement in implementing the Framework in practice. Encouraging involvement of family carers could potentially alleviate some of the demand on care home staff.<br><br>**Adaptation**: Family members should be actively engaged with, and offered the opportunity to be involved from the start e.g. through hosting a coffee morning or attending the monthly family meet up to promote the intervention	**Actor:** Intervention developer, care home staff (of all grades), family carers.<br><br>**Action:** Organise meetings with all potential users to explain what using the intervention would entail and what will be required both of the team and the individual. Meet with the senior and junior staff together to promote legitimacy and management approval of utilising the intervention. Hold coffee mornings/attend family member support groups to meet with family members of the residents to encourage their involvement.<br><br>**Target of the action:** To facilitate user engagement and collective commitment to implementing the intervention<br><br>**Temporality:** Following identification of sites, prior to implementation.<br><br>**Dose:** Multiple meetings should be scheduled to ensure all relevant people are reached (staff and family members) and accommodate for

			different shift patterns.<br><br>**Implementation outcome affected:** Adoption at staff level, reach within the care home and fidelity (the intervention being adhered to).<br><br>**Justification:** NPT (coherence and cognitive participation), CFIR (Inner setting: learning climate, leadership engagement, Individual characteristics: Knowledge and beliefs about an intervention, Implementation process: Engaging end-users), systematic review, co-design workshop and feasibility study data analysis.
	Access new funding		**Actor:** Intervention developer finance team<br><br>**Action:** To reimburse care homes for time taken to implement the intervention e.g. for site set-up meetings, staff training, ongoing additional duties<br><br>**Target of the action:** Providing resource and incentive to participate in the study and to promote staff engagement<br><br>**Temporality:** First payment should occur at the end of the first month<br><br>**Dose:** Once monthly throughout implementation<br><br>**Implementation outcome affected:** Adoption and staff and site level<br><br>**Justification:** NPT (cognitive participation, collective action), CFIR (Inner setting: Available resources), systematic review.
**<u>Implementation</u>**<br><br>**Strategies to ensure**	*Promote adaptability and tailor strategies*	- Whilst promoting adaptability of implementation strategies is key to ensure they can meet the needs of the user and context, this can lead	**Actor:** Intervention developers, all potential users<br><br>**Action:** To encourage flexibility in using the Framework, and to tailor the implementation strategies to the local needs of the care home e.g.

**individuals can enact the intervention**<br><br>**Targets NPT constructs: Collective Action**		to a lack of clarity surrounding the process and a lack of accountability for enacting the intervention.<br><br>**Adaptation**: Delineating individual's roles and responsibilities in advance is crucial to avoid confusion.	regularity of assessment, who conducted it.<br><br>**Target of the action:** To ensure that the Framework is compatible with existing care home practice, and ensure all individuals are aware of their roles and responsibilities.<br><br>**Temporality:** A meeting should be held prior to implementation, to learn about routine practice to minimise duplication of processes. Fortnightly catch ups with users thereafter.<br><br>**Dose:** Initial meeting one hour, 30 minute fortnightly follow up meetings.<br><br>**Implementation outcome affected:** Fidelity, feasibility and acceptability<br><br>**Justification:** NPT (collective action), CFIR (Intervention characteristics: adaptability, Inner setting: compatibility) systematic review, co-design workshop and feasibility study data analysis.
	Assess for readiness, need and identify barriers and facilitators	-If individuals do not perceive that there is a need to work differently or that something is lacking in the current process, it will result in users perceiving the Framework as superfluous.<br><br>- Particular consideration should be placed on ensuring the Framework is interoperable with any tablets already in use, to avoid multiple and/or unfamiliar devices.	**Actor:** Intervention developers, care home staff.<br><br>**Action:** To conduct an assessment of barriers and facilitators to uptake of the intervention, to determine readiness for change (55) and what support needs to be put in place e.g. provision of tablets, access to internet, training.<br><br>**Target of the action:** Providing care homes with the support they require to adopt and utilise the intervention, and to assess readiness and need for change<br><br>**Temporality:** This should occur at the same meeting where how to promote adaptability between the intervention and care home is

		**Adaptation**: Assessing care home need, as well as an assessment of readiness, is important to identify if it is an organisation which will be receptive to change.<br><br>It is essential these assessments are conducted is prior to implementation, strengthen the context analysis.	discussed. It is essential this is prior to implementation.<br><br>**Dose:** One hour meeting, prior to implementation<br><br>**Implementation outcome affected:** Adoption and feasibility of implementing the intervention<br><br>**Justification**: NPT (collective action), CFIR (Inner setting: Compatibility, Inner setting: Available resource), systematic review, co-design workshop and feasibility study data analysis.
	Remind practitioners	- Notifications built into the app are insufficient to initiate access.<br><br>**Adaptation**: Push notifications or alert via email to remind practitioners when an assessment is due is vital, especially in the early stages of implementation whilst attempting normalisation into routine practice.	**Actor:** Intervention developers<br><br>**Action:** To provide notifications to users to initiate opening the app when an assessment is due, to facilitate uptake.<br><br>**Target of the action:** Users are reminded when an assessment is due without having to log into the app to see inbuilt notifications, to facilitate uptake into routine practice<br><br>**Temporality:** The first reminder should be set for when the first assessment is due<br><br>**Dose:** As regularly as the assessment is due for the individual (suggested 1x a month minimum)<br><br>**Implementation outcome affected:** Adoption at staff level<br><br>**Justification:** NPT (collective action), co-design workshop and feasibility study data analysis.
	Identify and	- It is unlikely that staff members	**Actor:** Intervention developers, managers and staff champions

	prepare champions	who are delegated as champions will have sufficient time to perform their duties alongside their already demanding workload.<br><br>- Uptake of the Framework relied heavily on presence of a researcher undertaking the role of champion.<br><br>**Adaptation**: An external facilitator or staff member with full-time capacity to support implementation of eHealth should be employed in the early months.<br><br>What is required of a champion should be make explicit before individuals accept the role, to ensure they have capacity to undertake it.	**Action:** To identify champions within the home to monitor and drive implementation, deliver training to others and support other users. Champions receive additional training on app functionality and what is required in their role. Fortnightly meetings are held with the research team to monitor progress and refine strategies in response to barriers that arise.<br><br>**Target of the action:** Staff are supported in delivering the intervention, and barriers to uptake are iteratively responded to in real-time to facilitate uptake.<br><br>**Temporality:** Champions should be identified prior to implementation, so additional training can be delivered.<br><br>**Dose:** Champions are required throughout implementation. Fortnightly 30 minute meetings should be scheduled to monitor progress.<br><br>**Implementation outcome affected:** Intervention fidelity, feasibility, adoption and reach.<br><br>**Justification:** NPT (collective action, cognitive participation), CFIR (Implementation process: engaging champions), systematic review, co-design workshop and feasibility study data analysis.
	Conduct educational outreach visits	There is huge variation between the amount of training and support different staff members required, depending on their individual aptitudes and confidence, experience and familiarity with	**Actor:** Intervention developers, potential users<br><br>**Action:** To deliver thorough, personalised training to all potential users via multiple means, with a focus on the individual Framework components plus how they link together. eHealth literacy and prior experience should be considered, and refresher training offered as and when required. Training package should include:

		technology.<br><br>**Adaptation**: The face-to-face training session should be divided individual components - assessment and decision support functions - with a focus on the linkage between them. It should be delivered in 'bitesize' sections for each component, with a final session focusing on how they work together, with recorded narrated presentations provided.<br><br>-Training must also be tailored to the individual, with a consideration of the diversity of care home staff e.g. experience, individual aptitude, and e-literacy.<br><br>- Additional refresher training should be offered throughout implementation, and the original training should not be delivered too far in advance of study set up<br><br>- Family members should be offered training and if wanted, given access to the app to foster a more collaborative relationship	- A pack of training resources, user manuals, a glossary, a paper version of the intervention, contact details to access technical support (phone number and email).<br><br>- A face-to-face interactive training session divided into three 'bitesize' sections, relating to the IPOS-Dem, the decision support tools and how the components work together.<br><br>- A series of 'bitesize' instructional videos built into the app<br><br>- Certificates to be provided to staff on completion of training<br><br>**Target of the action:** Providing users with the knowledge and skills required to successfully enact the Framework<br><br>**Temporality:** The first training session should occur within one month of implementation. Refresher sessions to be offered weekly until no longer required.<br><br>**Dose:** One full face-to-face day session, followed by 30 min refresher sessions (or longer if required).<br><br>**Implementation outcome affected:** Staff adoption, acceptability, feasibility and fidelity of the intervention<br><br>**Justification:** NPT (collective action), CFIR (Inner setting: Access to knowledge), systematic review, co-design workshop and feasibility study data analysis.

		between them and care home staff.	
**<u>Sustainability</u>**<br><br>**Strategies to ensure individuals sustain use of the intervention**<br><br>**Targets NPT construct: Reflexive Monitoring**	*Audit and provide feedback*	- Although suggested, staff were not accessing functions on the app which allowed them to observe the impact of the Framework on residents' symptoms over time.<br><br>**Adaptation**: In future, automatic reports detailing resident outcomes each month should be generated and emailed to the staff members.	**Actor:** Intervention developers, care home staff<br><br>**Action:** To provide users with access to data pertaining to the impact of the app on resident symptoms over time. An automatic monthly report should be generated per resident and emailed to users.<br><br>**Target of the action:** Users can observe the effects and appraise the impact of the app with the intention it will motivate repeated use<br><br>**Temporality:** From the second assessment time point (likely month two)<br><br>**Dose:** A review of this data should be encouraged at every other champion meeting for 10 minutes.<br><br>**Implementation outcome affected:** Adoption at staff level, reach, acceptability and fidelity of the intervention.<br><br>**Justification**: NPT (reflexive monitoring) CFIR (Implementation Process: Reflecting and evaluating), systematic review, co-design workshop and feasibility study data analysis.
	Purposely and regularly re-examine the implementation	- Adaptations to the implementation plan should be recorded systematically via use of a Framework such as the FRAME-IS (56).	**Actor:** Intervention developers, champions<br><br>**Action:** To continuously monitor and review implementation strategies and iteratively adjust them in response to barriers to uptake, to encourage sustained use. Multiple methods should be utilised e.g. champion meetings, observations of the observations of the Framework in use to observe if anything is impeding uptake and analysis of app usage data.<br><br>**Target of the action:** Implementation strategies are responsive to real-

			time factors which are influencing uptake of the intervention<br><br>**Temporality:** The first champion meeting should take place as the study opens, to support set up of the app. App data should first be reviewed one month following implementation.<br><br>**Dose:** Champion meetings fortnightly for 30 minutes, app data review monthly, regular observations of the Framework in use (according to care home schedule).<br><br>**Implementation outcome affected:** Adoption, reach within the care home, acceptability, feasibility and fidelity of the intervention.<br><br>**Justification:** NPT (reflexive monitoring), CFIR (reflecting and evaluating), systematic review co-design workshop and feasibility study data analysis.
